# Health of the City

**Published:** 1845-08

**Authors:** 


### HEALTH OF THE CITY.

The health of Louisville was never better than it is at present. It would be difficult, indeed, to find anywhere a healthier city than it is. Hardly any cholera infantum has prevailed during the season, although for a few weeks we had a very high temperature, the thermometer rising occasionally as high as 96 deg. in the shade.

